# Impact of group housing of pregnant sows on health

**DOI:** 10.1186/s40813-016-0032-3

**Published:** 2016-07-01

**Authors:** Dominiek Maes, Liesbet Pluym, Olli Peltoniemi

**Affiliations:** 1 Unit of Porcine Health Management, Department of Reproduction, Obstetrics and Herd Health, Faculty of Veterinary Medicine, Ghent University, Merelbeke, Belgium; 2 Present address: Belpork vzw, Koning Albert II-laan 35 Bus 54, 1030 Brussels, Belgium; 3 Department of Production Animal Medicine, Faculty of Veterinary Medicine, University of Helsinki, Helsinki, Finland

**Keywords:** Sow, Gestation, Group housing, Health

## Abstract

Group housing of sows during gestation is mandatory in the EU since 2013. Compared to housing in individual crates, group housing allows the animals to express normal activity and behavior. The present paper discusses the impact of group housing on health, with emphasis on lameness, aggression and possible spread of infectious diseases. The prevalence of lameness is generally higher in sows housed in group than in sows housed individually. Floor space per sow, group size, pen design and flooring are the main factors of group housing involved in lameness development. Especially floor characteristics are important, and particular attention should be paid to the type, building material and quality of the floor, hygiene and the use of bedding such as straw or rubber mats. Aggression between sows is another critical issue in group housing systems. It occurs predominantly because of competition for access to a limited resource, or to establish a social hierarchy. Key factors to prevent aggression in group housing include gradual familiarization of unfamiliar animals, sufficient space and pen structure during initial mixing, minimizing opportunities for dominant sows to steal food from subordinates, provision of a good quality floor, environmental enrichment and use of straw bedding. Very scarce evidence-based information is available on the relationship between group housing and infectious disease. Compared to individual housing, sows in group housing have more nose-to-nose contact, and they have more oral contact with feces and urine. These factors could contribute to a higher or faster transmission of pathogens, but so far, there is no evidence showing more disease problems in group housing systems. In conclusion, in group housing systems, particular attention should be paid to prevention of lameness and aggression. Management is crucial but also feeding strategies, floor and bedding, and design of housing are very important as relatively minor adjustments may exert major effects on the animals.

## Background

Directive 91/630/EEC sets the minimum standards for the protection of pigs in the EU. It has been substantially amended several times. Directive 2008/120/EC [1] collates the existing legislation into one document. The most important items in this Directive in relation to the welfare of pregnant sows pertain to the necessity of group housing from 4 weeks after service to one week before expected farrowing, available space, floor characteristics, handling of diseased animals, provision and quality of food and drinking water and minimum requirements for light. An overview is shown in Table 1. Member states are allowed to be stricter than the EU legislation.

**Table 1:** Important items in the legislation (Directive 2008/120/EC) in relation to the welfare of pregnant sows

Parameter	Requirement
Minimum unobstructed floor space	Gilt after service >1.64 m^2^
	Sows and gilts in group >2.25 m^2^
	<6 animals: +10 %
	>40 animals: −10 %
Continuous solid floor for pregnant gilts and sows	>0.95 m^2^ per gilt; >1.3 m^2^ per sow
Drainage opening	Max. 15 %
Slats for pregnant gilts and sows	Gap width: max. 20 mm
	Slat width: min. 80 mm
Group housing	Mandatory from 4 weeks after service to one week before expected farrowing
Manipulable material	Permanent access for sows and gilts
Food	Sufficient bulky or high-fibre food as well as high-energy food for each individual
Feeding	At least once per day
Drinking water	Permanent access to fresh water
Diseased/injured pigs in group housing	May be housed individually in sick bay; should be able to turn around
Continuous noise levels	<85 dB
Amount of light	>40 lux for min. 8 h/day
Lying area	Comfortable, all animals should be able to use it simultaneously

Many different group housing systems are used for pregnant sows. Differences mainly relate to type of feeding system (individual versus group feeding, restricted versus *ad libitum* feeding, physical separation of the sows during feeding or not, simultaneous versus sequential feeding), group characteristics (dynamic versus stable, size of the group), type of floor and presence of bedding material. The present paper does not aim to review the advantages and disadvantages of the different systems, but to discuss the impact of group housing of pregnant sows on health, with emphasis on lameness, aggression and possible spread of infectious diseases. Comparison will be made mainly with housing sows in individual crates. The effects of group housing on sow production and reproduction is discussed in an accompanying paper [2].

## Review

### Group housing and lameness

Lameness can be defined as impaired movement or deviation from normal gait or posture while a normal degree of alertness is displayed [3]. The severity of lameness can vary greatly. Lameness may be manifested as a decreased symmetry of limb movement, an alteration or shortening of stride, a reduced ability or inability to bear weight and total recumbency. Prevalence rates ranging from 6 to 17 % have been reported [4–6]. Lameness is economically very important. The cost of lameness has been estimated to vary from €37 to €133 per lame sow [7].

The prevalence of lameness is generally higher in sows housed in group than in sows housed individually [8, 9], although this is not always the case [10] as many other factors (e.g. management, feeding, type of floor) apart from the housing system may also determine the occurrence of lameness problems. Housing may affect the risk of lameness development either directly through its impact on the components of the locomotor system, or indirectly by influencing the number and type of movements sows can make. Floor space per sow, group size, pen design and flooring are the main components of group-housing that may be involved in lameness development.

### Floor space, group size and pen design

In a study by Salak-Johnson et al. [11], three static social groups of five sows per group were assigned to 1.4, 2.3 and 3.3 m^2^ of floor space per sow, respectively. Sows were fed using floor feeding. No space effect on lameness could be found within the first 13 days of group-housing but in the weeks thereafter, a high space allowance was related to an increased risk of lameness. Animals allocated to a high floor space may have had greater opportunity for activity which may have increased the risk of injuries leading to lameness. The effect of space allowance on lameness development may also be dependent on group size. In finishing pigs, restricted floor space resulted in greater lameness scores in large groups (*n* = 108) but reduced scores in small groups (*n* = 18) [12]. The group size used by Salak-Johnson et al. [11] was rather small (*n* = 5) and may explain why the higher space allowance increased the risk of sow lameness in their study.

Furthermore, studies on aggressive and social behaviour of sows revealed that space requirements not only differ according to group size but also to group stability, pen design and feeding system [13]. This may also apply to lameness development. Therefore, care must be taken when attempting to generalize the results found by Salak-Johnsosn et al. [11]. Differences in herd-related factors could have been the reason why Gjein and Larssen [14] and Heinonen et al. [10] did not find a significant association between space allowance and lameness in group-housed sows. The minimal space according to EU legislation applies to all group-housing systems regardless of feeding system, group stability or use of bedding though adjustments are made with regard to group size. For sows housed in dynamic social groups and fed by an electronic sow feeder, 33 % more area than the EU legal minimum was demonstrated to induce better welfare considering agonistic behaviour and consecutive wounds [15]. The impact on productivity and social physiological stress was not yet determined. Nonetheless, these results may indicate that the legal minimum requirements may be insufficient for sows housed in dynamic groups and fed by electronic sow feeders. Table 2 shows an overview of the main feeding systems for group-housed pregnant sows. As can be seen, physical separation during feeding is complete in electronic feeding systems with sow identification and in free access stalls. Sows can be fed with an individual ration only in electronic feeding systems with sow identification, and sows can eat simultaneously in free access stalls, and in drop or floor feeding systems. It is clear that many factors of the housing system are interrelated, influence how sows interact with each other, and consequently influence the risk for lameness.

**Table 2:** Commonly used feeding systems for group-housed pregnant sows

Feeding system	Physical separation during feeding	Individual ration	Eating simultaneously
Electronic feeding station with sow identification	complete	yes	no
Electronic feeding station without sow identification	no	no	no
Free access stalls	complete	no	yes
Drop feeding	partial (no)	no	yes
Floor feeding	no	no	yes
*Ad libitum* feeding	no	no	no

### Floor characteristics

The type, building material and quality of the floor, hygiene and the use of bedding may all influence the risk of lameness.

#### Type of floor

Three main floor types are used for pigs, namely solid, partially slatted and fully slatted floors. Use of slatted, unbedded floors increases the risk for lameness in group-housed gestating sows. The odds of being lame on slatted floors is twice as high compared to non-slatted floors [10], 4.6 times higher compared to solid concrete floors with deep bedding and 4.8 times higher than in sows housed outdoors [16]. The association between floor type and lameness might occur as a result of an increased prevalence of locomotor disorders or because locomotor disorders are more painful or cause more discomfort. On slatted floors, pigs may face some challenges such as chipped, sharp or worn slat edges [17], slipping and wedging the claws into the voids [18], and an increased, uneven distribution of pressure on the claws [17, 18]. These effects suggest that slatted floors cause lameness more likely by affecting claw health than by affecting joint soundness.

#### Building material

Concrete is the most commonly used floor material for housing pregnant sows. Gjein and Larssen [14] showed that the risk for lame sows on concrete slats was 2.4 times higher than on plastic slats. Claw lesions did not differ between both materials indicating that the difference in lameness may have been due to other locomotor disorders or that claw lesions may have been less painful when walking on plastic slats [19]. Plastic slats are softer and may result in less biomechanical stress to the claws and limbs compared with concrete slats [14, 19]. Hence, plastic slats may be more comfortable to walk on and mildly lame sows may be missed when scoring sows on plastic slats. However, plastic slats can be slippery [19], and possibly also predispose to hoof overgrowth. So far, no clear-cut advice on alternatives to concrete slats can be given to reduce the risk of lameness development in group-housed sows. Therefore, further research is warranted.

#### Quality of the floor

Slip-resistance, abrasiveness, surface profile and hardness are the four main quality factors contributing to a floor’s total injury potential [20]. Floors with a low slip-resistance may result in slipping and falling causing skin abrasions, bruising, bursitis, torn dewclaws and even broken bones [17, 21]. Pigs may adapt their gait to potentially slippery surfaces by reducing walking speed, shortening stride length, prolonging stance time and by employing more three-limb support phases. However, when slip-resistance becomes too low, adaption might no longer be sufficient. To prevent pigs from slipping on solid concrete floors, a floor coefficient of friction of at least 0.63 has been determined [22]. Floors with a high abrasion level predispose to calluses [23] as well as skin wounds and claw lesions [24]. Especially hoof cracks have been associated with abrasive floors. A minimum of abrasiveness however, is necessary to prevent hoof overgrowth [20]. Using slatted floors, also the void ratio (i.e. area of holes per unit area of floor), slat width and edge design should be taken into account. Hard surfaces e.g. concrete may lead to pressure injuries. On soft surfaces, e.g. soil or rubber mats, the area of contact between the claw and the floor is higher. This may reduce the overloading of claws [25]. Finally, durability of the floor is important. A worn surface can be injurious and is difficult to clean and disinfect. De Belie [26] found that concrete slats could already show degradation within five years leading to increased surface roughness, enlarged gaps and animal injuries. These claw or limb lesions may result in clinical lameness [27], but studies directly associating clinical lameness with floor quality have not been performed yet.

#### Hygiene

Floors should be dry and clean. In pens with dirty, wet slatted floors, the risk of lameness is 2.8 times higher than in pens with good floor hygiene [14]. The increased risk is assumed to be mainly claw related. An increased risk of, particularly, heel/sole erosions on dirty and wet floors has been reported for finishing pigs [28] and lactating sows [29]. In group-housed sows, heel overgrowth seemed to be more common in herds with poor floor hygiene [30]. Softening of the hoof horn, a higher slipperiness of the floor and the acidic caustic nature of slurry may contribute to the increased incidence of claw lesions on wet, slurry covered floors. Also, dirty floors may cause infection of claw lesions enhancing lameness. Gjein and Larssen [19] reported a 4.2 times higher risk of claw infections for group-housed sows in herds with poor floor hygiene.

#### Use of bedding

Use of solid concrete covered with deep straw bedding has been associated with a lower prevalence of lameness compared to bare solid and slatted floors [29]. This might be explained by a reduced prevalence of locomotor disorders or because deep bedding is more comfortable to sows. Housing sows during gestation on solid concrete with straw bedding decreased the risk of heel flaps but increased the risk of toe erosions during the subsequent lactation [29]. Toe erosions may be more prevalent on straw bedding, likely due to the lack of natural wearing. When straw bedding is used, hygiene is important. Damp straw, soiled with feces and urine softens the hooves, makes them more prone to abrasion and pressure-induced lesions and increases the risk of claw infections and hence lameness. Provision of straw has not been universally adopted as it is not compatible with slatted floors and liquid manure handling systems. In addition, it can be costly and labour intensive [31].

Rubber mats have been suggested to be a useful alternative for straw [32, 33]. Elmore et al. [32] could not find a difference in lameness scores between sows housed on rubber mats versus concrete floors. However, in that study, rubber mats were only added to the feeding stalls and not to the area with concrete slatted flooring between the stalls. By contrast, Díaz et al. [33] covered the concrete slatted flooring in both the feeding stalls and the group area with rubber slat mats. Sows housed on rubber slat mats had a significantly reduced risk of becoming lame. Bos et al. [34] investigated the effect of a rubber top layer on both the slatted and unslatted areas of the pens’ floor compared to conventional concrete floors on sows’ gait. From four weeks after insemination until one week before parturition, three groups were housed in pens with concrete floors (40.3 m^2^ slatted and 31.7 m^2^ solid), and three groups in identical pens but with a rubber top layer fitted to all slatted and half the solid floor. For both floor types, the prevalence of lameness increased during gestation and decreased during lactation. However, the sows’ gait was significantly better at the end of the gestation phase when housed on rubber-topped floors (difference: 9.88 mm on 150 mm tagged visual analogue scale), indicating that a rubber topped-floor may increase the likelihood of healing lameness.

On rubber mats, the contact area between the claw and the floor is larger improving the claw pressure distribution and reducing overloading of claws and joints [25]. This cushioning effect may also improve circulation in the foot [35]. Finally, rubber slat mats may provide more traction and hence reduce lameness due to slipping [36]. Besides the potential to reduce the risk of lameness, rubber slat mats have also been associated with an increased risk of toe overgrowth, heel-sole cracks, white line lesions and wall cracks [33].

Apart from straw and rubber mats, floor substrates such as peat, mushroom compost, and wood chips may better resemble natural lying surfaces and be more attractive to sows [37]. They could better allow for rooting behavior, control of the thermal environment, reduce lameness, and cause less problems for manure management systems. These substrates however have not been well studied in group housing systems for sows.

### Group housing and aggression

Aggression occurs predominantly because of competition for access to a limited resource, or to establish a social hierarchy. Aggression related to competition for feed is generally short in duration, but very frequent. If aggression is related to establishing a social hierarchy, it is less frequent, but can be far more intense. Social hierarchy is established within 2 to 10 days after mixing of unfamiliar animals. Full integration of new sows in a resident group takes a lot longer. When small groups of gilts or sows were introduced in large dynamic groups, integration time was estimated to take at least 3–4 weeks [38]. In general, older sows are more dominant.

Most of the lesions caused by aggression are scratches or cuts on the skin. Vulva biting is also possible, but this is more regarded as a sign of frustration instead of aggression. Aggression may also result in lameness. Persistent aggression can decrease welfare as indicated by increased stress hormone concentrations [39], increased heart rates [40], increased injuries, and restricted access to resources [41] in animals that are aggressive or ones that are being attacked.

Many of the aspects discussed above (see lameness) also apply to prevent aggression. Key factors to prevent aggression include a gradual familiarization of unfamiliar animals, sufficient space and pen structure during initial mixing (less aggression in rectangular pens than in squared pens), minimizing opportunities for dominant sows to steal food from subordinates, the provision of a good quality floor, environmental enrichment and the use of straw bedding [42, 43]. Stevens et al. [44] found less aggression when mixing sows at day 35 of gestation compared to mixing them between day one and seven post-insemination. In a similar study, Knox et al. [45] observed the poorest reproductive performance and welfare when sows were mixed 3 to 7 days after breeding. There were few differences between the day 14 and day 35 treatments in reproduction or welfare, but day14 (not D35) differed from sows housed in individual stalls. Also the composition of the diet may influence the risk for aggression. Fibre rich diets help to create satiation and promote social stability and rest, when fed *ad libitum* [43]. Poletto et al. [46] showed that a diet enriched with tryptophan provided to gestating sows for a short period prior to social mixing and continued for a short time after is an effective means of reducing aggression and improving the welfare of sows during group formation. A reduction of feeding level, however, at day 17 of pregnancy considerably increased loss of embryos and the whole litter in group housed early pregnant gilts [47]. This study showed how sensitive gilts can be to reduction of feeding level during early pregnancy. The loss of embryos and the whole litter was suggested to be associated with increased stress due to increased competition within the group for reducing resources [47]. The presence of a boar may reduce the level of aggression between mixed sows, and decrease the intensity and frequency of fighting [48]. However, the effectiveness of boar presence may reduce with time, with aggression reduced only temporarily at or shortly after mixing. Plush et al. [49] demonstrated that the provision of pig appeasing pheromones significantly reduced the length of an aggressive event between two sows on the day of mixing although there were no significant effects on sow injury, cortisol concentrations or reproduction. Further research however needs to be carried out to assess its usefulness under commercial conditions. Sedation is an obvious but controversial choice in reducing aggression in sows. Overall, sedation seems to delay aggression rather than reduce it, in a similar manner to boar exposure or night mixing. Some reports suggest that aggression may also be managed through selection of pigs [50].

### Group housing and disease transmission

Very scarce evidence based information is available on the relationship between group housing and disease. Animals that are stressed are more susceptible to disease, may shed more pathogens e.g. *Salmonella* [51], and may be at higher risk for the development of gastric ulcers [52]. In general, cortisol levels in sows that are housed in groups are similar or lower than in sows housed individually [53]. Von Borell et al. [54], McGlone et al. [55] and Broom et al. [56] found that antibody production and neutrophil-to-lymphocyte ratio were not different between sows kept individually compared with those kept in groups.

Compared to housing sows in individual crates, sows in group housing have more nose-to-nose contact, and they have more oral contact with feces and urine. These factors could contribute to a higher or faster transmission of pathogens. However, also other aspects should be considered. Regarding respiratory disease, one could expect slightly better air quality in group housing systems because of a potential better ventilation of the entire stable. However, if hygiene is poor, ammonia levels can also be high. If straw is used, there will be more dust, but likely lower stress levels [57]. Because of more contact with feces, one can expect a higher risk for infections with intestinal pathogens such as *Brachyspira* spp., *Lawsonia intracellularis*, *Salmonella*, *Ascaris suum*. Reports [58, 59] showed higher seroconversion rates for *L. intracellularis* in group housing systems with straw bedding. The hygiene levels are largely dependent on the housing conditions and the management. The fact that the lying and defecation area are separated improves hygiene and decreases the intensity of oral contact with feces. Sows in group housing can move around, potentially leading to less problems with gastro-intestinal torsions. Movement is also beneficial to avoid constipation and urine stasis. Less problems with constipation may reduce the risk for periparturient dysgalactia syndrome [60]. Effects of group housing and stress on sow production and reproduction are discussed in the accompanying paper [2].

## Conclusion

In contrast with housing sows in individual crates, group housing allows the animals to express normal activity and behavior. However, group housing as such does not automatically imply better animal welfare. Particular attention should be paid to prevention of lameness and aggression as these may severely compromise welfare. Optimal functioning of group housing systems depends on the combined effect of different factors. Management is a crucial factor and it should be animal oriented. In addition, feeding strategies, floor and bedding, design of housing and their interactions are very important, as relatively minor adjustments may have major effects on the animals.

## References

[1] European Commission. Council Directive 2008/120/CE of 18 December 2008 laying down minimum standards for the protection of pigs. Retrieved from http://eur-lex.europa.eu/LexUriServ/LexUriServ.do?uri=OJ:L:2009:047:0005:0013:EN:PDF. Accessed 2 Sept 2015.

[2] Peltoniemi O, Björkman S, Maes D. Reproduction of group-housed sows. Porcine Health Management. 2016. In press. 10.1186/s40813-016-0033-2 PMC5382509 28405441

[3] Maas J, Smith BP (2009). Musculoskeletal abnormalities. Large Animal Internal Medicine.

[4] D’Eath R (2012). Repeated locomotion scoring of a sow herd to measure lameness: consistency over time, the effect of sow characteristics and inter-observer reliability. Anim Welfare.

[5] Pluym L, Van Nuffel A, Dewulf J, Cools A, Vangroenweghe F, Van Hoorebeke S, Maes D (2011). Prevalence and risk factors of claw lesions and lameness in pregnant sows in two types of group housing. Vet Med.

[6] Pluym L, Van Nuffel A, Van Weyenberg S, Maes D (2013). Prevalence of lameness and claw lesions during different stages in the reproductive cycle of sows and the impact on reproduction results. Animal.

[7] Deen J, Anil S, Anil L, Baidoo S (2008). Lameness overview and awareness: implications for welfare, housing, performance and economics. Proceedings of the FeetFirst™ European Symposium on Sow Lameness, Asten/Sterksel, The Netherlands.

[8] Chapinal N, Ruiz de la Torre J, Cerisuelo A, Gasa J, Baucells M, Coma J, Vidal A, Manteca X (2010). Evaluation of welfare and productivity in pregnant sows kept in stalls or in 2 different group housing systems. J Vet Behav.

[9] Estienne M, Harper A, Knight J (2006). Reproductive traits in gilts housed individually or in groups during the first thirty days of gestation. J Swine Hlth Prod.

[10] Heinonen M, Oravainen J, Orro T, Seppä-Lassila L, Ala-Kurikka E, Virolainen J, Tast A, Peltoniemi O (2006). Lameness and fertility of sows and gilts in randomly selected loose-housed herds in Finland. Vet Rec.

[11] Salak-Johnson J, Niekamp S, Rodriguez-Zas S, Ellis M, Curtis S (2007). Space allowance for dry, pregnant sows in pens: Body condition, skin lesions, and performance. J Anim Sci.

[12] Street B, Gonyou H (2008). Effects of housing finishing pigs in two group sizes and at two floor space allocations on production, health, behavior, and physiological variables. J Anim Sci.

[13] Weng R, Edwards S, English P (1998). Behaviour, social interactions and lesion scores of group-housed sows in relation to floor space allowance. Appl Anim Behav Sci.

[14] Gjein H, Larssen R (1995). The effect of claw lesions and claw infections on lameness in loose housing of pregnant sows. Acta Vet Scand.

[15] Remience V, Wavreille J, Canart B, Meunier-Salaün M, Prunier A, Bartiaux-Thill N, Nicks B, Vandenheede M (2008). Effects of space allowance on the welfare of dry sows kept in dynamic groups and fed with an electronic sow feeder. Appl Anim Beh Sci.

[16] KilBride A, Gillman C, Green L (2009). A cross-sectional study of the prevalence of lameness in finishing pigs, gilts and pregnant sows and associations with limb lesions and floor types on commercial farms in England. Anim Welfare.

[17] KilBride A, Gillman C, Ossent P, Green L (2009). Impact of flooring on the health and welfare of pigs. In Practice.

[18] Mouttotou N, Hatchell F, Green L (1999). Foot lesions in finishing pigs and their associations with the type of floor. Vet Rec.

[19] Gjein H, Larssen R (1995). Housing of pregnant sows in loose and confined systems A field study 3. The impact of housing factors on claw lesions. Acta Vet Scand.

[20] McKee C, Dumelow J (1995). A review of the factors involved in developing effective non-slip floors for pigs. J Agric Eng Res.

[21] Dewey C, Straw BE, Zimmerman JJ, D’Allaire S, Taylor DJ (2006). Diseases of the nervous and locomotor systems. Diseases of Swine.

[22] Thorup V, Tøgersen F, Jørgensen B, Jensen B (2007). Biomechanical gait analysis of pigs walking on solid concrete floor. Animal.

[23] Leeb B, Leeb C, Troxler J, Schuh M (2001). Skin lesions and callosities in grouphoused pregnant sows: animal-related welfare indicators. Acta Agr Scand, Section A-Anim Sci.

[24] Geyer H, Troxler J (1988). Hoof diseases as a result of stall floor defects. Tierärztl Praxis.

[25] Carvalho V, de Alencar Nääs I, Mollo Neto M, Souza S (2009). Measurement of pig claw pressure distribution. Biosyst Eng.

[26] De Belie N (1997). A survey on concrete floors in pig houses and their degradation. J Agr Eng Res.

[27] Webb N, Nilsson C, Baxter SH, Baxter MR, McCormack JAC (1983). Flooring and injury − an overview. Farm Animal Housing and Welfare.

[28] Penny R, Osborne A, Wright A, Stephens T (1965). Foot-rot in pigs: Observations on the clinical disease. Vet Rec.

[29] Kilbride A, Gillman C, Green L (2010). A cross-sectional study of prevalence and risk factors for foot lesions and abnormal posture in lactating sows on commercial farms in England. Anim Welfare.

[30] Gjein H, Larssen R (1995). Housing of pregnant sows in loose and confined systems A field study 2. Claw lesions: Morphology, prevalence, location and relation to age. Acta Vet Scand.

[31] Whittaker X, Edwards S, Spoolder H, Lawrence A, Corning S (1999). Effects of straw bedding and high fibre diets on the behaviour of floor fed group-housed sows. Appl Anim Beh Sci.

[32] Elmore M, Garner J, Johnson A, Richert B, Pajor E (2010). A flooring comparison: The impact of rubber mats on the health, behavior, and welfare of group-housed sows at breeding. Appl Anim Beh Sci.

[33] Díaz J, Fahey A, KilBride A, Green L, Boyle L (2013). Longitudinal study of the effect of rubber slat mats on locomotory ability, body, limb and claw lesions, and dirtiness of group housed sows. J Anim Sci.

[34] Bos EJ, Maes D, van Riet M, Millet S, Ampe B, Janssens G, Tuyttens F (2015). Effect of rubber top layer on concrete floors on gait score in group housed sows. Proc. International conference on pig welfare: Improving Pig Welfare - what are the ways forward? Copenhagen Denmark.

[35] Singh S, Ward W, Lautenbach K, Hughes J, Murray R (1993). Behaviour of first lactation and adult dairy cows while housed and at pasture and its relationship with sole lesions. Vet Rec.

[36] Boyle L, Regan D, Leonard F, Lynch P, Brophy P (2000). The effect of mats on the welfare of sows and piglets in the farrowing house. An Welfare.

[37] Bench C, Rioja-Lang F, Hayne S, Gonyou H (2013). Group gestation sow housing with individual feeding – II: How space allowance, group size and composition, and flooring affect sow welfare. Livest Sci.

[38] Spoolder H, Lawrence A, Edwards S, Simmins P, Armsby A (1998). The effects of food level on the spatial organization of dynamic groups of sows. Proceedings of the Annual Meeting of the International Society for Applied Ethology, Clermont-Ferrand, France.

[39] Otten W, Puppe B, Kanitz E, Schön P, Stebenow B (1999). Effects of dominance and familiarity on behaviour and plasma stress hormones in growing pigs during social confrontation. J Vet Med.

[40] Marchant J, Mendl M, Rudd A, Broom D (1995). The effect of agonistic interactions on the heart rate of group-housed sows. Appl Anim Beh.

[41] O’Connell N, Beattie V, Moss B (2003). Influence of social status on the welfare of sows in static and dynamic groups. Anim Welfare.

[42] Greenwood E, Plush K, van Wettere W, Hughes P (2014). Hierarchy formation in newly mixed group housed sows and management strategies aimed at reducing its impact. Appl Anim Beh Sci.

[43] Spoolder H, Geudeke M, Van der Peet-Schwering C, Soede N (2009). Group housing of sows in early pregnancy: a review of success and risk factors. Livest Sci.

[44] Stevens B, Karlen G, Morrison R, Gonyou H, Butler K, Kerswell K, Hemsworth P (2015). Effects of stage of gestation at mixing on aggression, injuries and stress in sows. Appl Anim Beh Sci.

[45] Knox R, Salak-Johnson J, Hopgood M, Greiner L, Connor J (2014). Effect of day of mixing gestating sows on measures of reproductive performance and animal welfare. J Anim Sci.

[46] Poletto R, Kretzer F, Hötzel M (2014). Minimizing aggression during mixing of gestating sows with supplementation of a tryptophan-enriched diet. Physiol Behav.

[47] Virolainen J, Tast A, Sorsa A, Love R, Peltoniemi O (2004). Changes in feeding level during early pregnancy affects fertility in gilts. Anim Reprod Sci.

[48] Borberg C, Hoy S (2009). Mixing of sows with or without the presence of a boar. Livest Sci.

[49] Plush K, Herde P, Rajapakse U, Hughes P, van Wettere W (2013). Pig appeasing pheromones reduce duration of aggression butnot cortisol levels in newly-mixed gestating sows.

[50] Erhard H, Mendl M, Ashley D (1997). Individual aggressiveness of pigs can be measured and used to reduce aggression after mixing. Appl Anim Behav Sci.

[51] Verbrugghe E, Boyen F, Van Parys A, Van Deun K, Croubels S, Thompson A, Shearer N, Leyman B, Haesebrouck F, Pasmans F (2011). Stress induced *Salmonella* Typhimurium recrudescence in pigs coincides with cortisol induced increased intracellular proliferation in macrophages. Vet Res.

[52] Van der Meulen J, de Graaf G, Nabuurs M, Niewold T. Effect of transportation stress on intramucosal pH and intestinal permeability. In: JE Lindberg, B Ogle, Editors. Digestive physiology of pigs. Wallingford, UK: CAB International. 2001. p. 329–31.

[53] McGlone J, von Borell E, Deen J, Johnson A, Levis D, Meunier-Salaün M, Morrow J, Reeves D, Salak-Johnson J, Sundberg P (2004). Review: compilation of the scientific literature comparing housing systems for gestating sows and gilts using measures of physiology, behaviour, performance and health. Prof Anim Sci.

[54] Von Borell E, Morris J, Hurnik J, Mallard B, Buhr M (1992). The performance of gilts in a new group housing system: endocrinological and immunological functions. J Anim Sci.

[55] McGlone J, Salak-Johnson J, Nicholson R, Hicks T (1994). Evaluation of crates and girth tethers for sows: reproductive performance, immunity, behavior and ergonomic measures. Appl Anim Behav Sci.

[56] Broom D, Mendl M, Zanella A (1995). A comparison of the welfare of sows in different housing conditions. Anim Sci.

[57] Tuyttens F (2005). The importance of straw for pig and cattle welfare: a review. Appl Anim Behav Sci.

[58] Chouet S, Prieto C, Mieli L, Veenhuizen M, McOrist S (2003). Patterns of exposure to *Lawsonia intracellularis* infection on European pig farms. Vet Rec.

[59] Stege H, Jensen T, Möller K, Verstergaard K, Baekbo P, Jorsal S (2000). Infection dynamics of Lawsonia intracellularis in Danish pig herds. Prev Vet Med.

[60] Papadopoulos G, Vanderhaeghe C, Janssens G, Dewulf J, Maes D (2010). Risk factors associated with postpartum dysgalactia syndrome in sows. Vet J.

